# Sulphide of Calcium

**Published:** 1876-07

**Authors:** T. Curtis Smith

**Affiliations:** Middleport, Ohio


### SULPHIDE OF CALCIUM.

By T. CURTIS SMITH, M.D., Middleport, Ohio.

I take pleasure in calling attention to this remedy for action in a field where an agent that will produce the effects claimed for it is much needed, viz.: in discussing glandular swellings which threaten suppuration, in furuncular eruptions, carbuncles, and chronic induration of various tissues, the result of inflammatory action. Sidney Ringer, of London, has done much to bring this prominently before the profession as a valuable therapeutic agent. He, however, signally fails to give the rationale of its mode of action upon the physical economy, further than to claim great alterative action for it. This is but another method of covering up our ignorance of the real actions of various drugs now in general use.

I have used this agent more or less for a year past, and am well satisfied that it possesses some valuable properties for difficulties of the classes above named.

In cases where glandular abscesses are threatened in scrofulous children, it has the effect to hasten their removal by either rapidly increasing their tendency to suppuration and rapid healthy granulation, or of removing them quickly by absorption without suppuration. This seems like a contradiction of terms, but we may well remember that other and apparently less potent agents have similar therapeutic value. Very often warm moist applications are used to assist in alleviating inflammation, and the same kind of applications are also used to hasten suppuration. So we claim no more for this agent than has long been practically claimed in these different actions for a common poultice, but we hold that it is far more efficient.

We not unfrequently meet with glandular swellings in strumous children and adults, not of specific origin. These often become very large. Sometimes, after weeks of suffering and inconvenience from the swellings, they suppurate, and after opening are very slow to heal, and for weeks will discharge an unhealthy looking pus; and when they have healed, too often leave very ugly cicatrices about the neck. If in such cases the sulphide of calcium is given, and without any other aid, these swellings, however large they may be, will soon disappear, unless suppuration has already begun. In the latter case, instead of a long, tedious course of waiting through days and nights of suffering, and increased general debility, they will rapidly hasten to a termination by the suppurative process. The pus formed under its use seems of a more laudable character than that commonly observed in strumous abscesses, and after being dischargd, healthy granulations spring up; soon the swelling has disappeared, and the point of opening has closed, leaving very little trace of the former trouble. If no pus has already formed in these large, threatening, indurated glands, then under the sulphide of calcium they soon become less in size, and in a remarkably short time disappear.

Another use of no mean value for this agent is in the treatment of boils. Several times in the last year have I met with cases where a succession of crops of furuncles would appear. As one crop would commence to decline another would put in an appearance. In all of such cases, where the sulphide of calcium has been resorted to, they have speedily recovered. The boils already formed would quickly suppurate, and discharge profusely, the "core" melting away with astonishing rapidity.

The little boils just commencing to form would rapidly disappear without ever becoming painful, and no trace of them could be observed. The points were notably gained in the case of an old lady who was so much annoyed from the boils that she could not sleep day or night, nor move in any direction without pain, often severe. In a few days she was relieved completely. Also in a recent case where a succession of these painful abscesses had occurred, those present rapidly disappeared, and have not yet returned.

In carbuncles I have had a more limited experience. Sidney Ringer is loud in his praise of the remedy in this very painful affection, and especially mentions the rapid solution of the indurated "core," and the formation and discharge of healthy pus, followed by rapid granulation, and not followed by a repetition of the swellings at other points.

In those children that are, from some constitutional vice, subject to a constant repetition of ill-conditioned sores, often greatly annoying them, and, from their very slow tendency to heal, last for weeks and months, the sulphide of calcium will soon effect a rapid recovery and prevent a tendency to their return.

I have recently used this remedy in a few cases of visceral indurations of long standing, with the effect of a rapid removal of the morbid condition and a speedy improvement in the general health. I am now trying its influence over some very intractable diseases, but cannot speak yet of its power in these new directions.

I have noticed, in the use of this agent, that there has been a general improvement in the health and vigor, and that especially the nervous system seemed to become more efficient, and through this and its alterative power, there would be a general improvement in the secretions; the skin and kidneys would act more freely; the bowels, if costive, would become at once regular; any tendency to acidity of the stomach would be partially or completely removed. I believe its influence in toning the nervous system is an effect fully as important as its alterative power. Time and an extensive use of this agent will decide whether it will maintain the high stand it now seems to claim.

Ringer claims the same effect for the sulphides of potassium and sodium. I have only used the sulphide of calcium. Dr. Ringer recommends doses of one-tenth of a grain repeated every two hours. It has been my habit to give to young children a half grain every four to six hours, and to an adult, from a half to two grains in sugar four to six times a day. Its odor is disagreeable, and similar to that given off by the Blue Lick mineral waters of Kentucky, and not unlike sulphuretted hydrogen. The taste, however, is easily concealed by mixing with sugar or making into pill. The solution in water cannot well be attained, and if it could, it would be very unpleasant to the patient to take.

I have no reason to believe the agent would have any special power over syphilitic indurations of any kind, but have not extended the use of the agent in this direction to ascertain what power it might exert, except in a single case, where it was used in connection with mercury. The results weie good, but whether the agent had any influence or not, I cannot say.

